# Effectiveness and safety of interspinous spacer versus decompressive surgery for lumbar spinal stenosis: A meta-analysis of randomized controlled trials

**DOI:** 10.1097/MD.0000000000036048

**Published:** 2023-11-17

**Authors:** Jian-Hai Xin, Jia-Ju Che, Zhe Wang, Yu-Ming Chen, Bing Leng, Da-Lin Wang

**Affiliations:** a Department One of Orthopedics, Affiliated Hospital of Beihua University, Jilin, China.

**Keywords:** decompressive surgery, interspinous spacer, lumbar spinal stenosis, meta-analysis, randomized controlled trials

## Abstract

**Study design:**

A meta-analysis of randomized controlled trials.

**Objective:**

Our meta-analysis was conducted to investigate whether interspinous spacer (IS) results in better performance for patients with lumbar spinal stenosis (LSS) when compared with decompressive surgery (DS).

**Background data:**

DS and IS are common surgeries for the treatment of LSS. However, controversy remains as to whether the IS is superior to DS.

**Methods:**

We comprehensively searched PubMed, EMBASE, and Cochrane Central Register of Controlled Trials for prospective randomized controlled trials that compared IS versus DS for LSS. The retrieved results were last updated on July 30, 2023.

**Results:**

Eight studies involving 852 individuals were included in the meta-analysis. The pooled data indicated that IS was superior to DS considering shorter operation time (*P* = .003), lower dural violation rate (*P* = .002), better Zurich Claudication Questionnaire Physical function score (*P* = .03), and smaller foraminal height decrease (*P* = .004), but inferior to DS considering the higher rate of reoperation (*P <* .0001). There was no significant difference between the 2 groups regarding hospital stay (*P* = .26), blood loss (*P* = .23), spinous process fracture (*P* = .09), disc height decrease (*P* = .87), VAS leg pain score (*P* = .43), VAS back pain score (*P* = .26), Oswestry Disability Index score (*P* = .08), and Zurich Claudication Questionnaire symptom severity (*P* = .50).

**Conclusions:**

In summary, we considered that IS had similar effects with DS in hospital stay, blood loss, spinous process fracture, disc height decrease, VAS score, Oswestry Disability Index score, and Zurich Claudication Questionnaire Symptom severity, and was better in some indices such as operation time, dural violation, Zurich Claudication Questionnaire Physical function, and foraminal height decrease than DS. However, due to the higher rate of reoperation in the IS group, we considered that both IS and DS were acceptable strategies for treating LSS. As a novel technique, further well-designed studies with longer-term follow-up are needed to evaluate the effectiveness and safety of IS.

## 1. Introduction

Lumbar spinal stenosis (LSS) is a common disease due to a degenerative decrease in the lumbar spinal complex.^[1,2]^ Decompression surgery (DS) is the standard surgical treatment for LSS, including wide laminectomy, segmental bilateral laminotomies, unilateral hemilaminectomy, or minimally invasive DS.^[3,4]^ Extensive decompression with partial or complete facetectomy can provide considerable relief from symptoms attributable to stenosis but may result in iatrogenic instability.^[5]^ Therefore, a minimally invasive and low-risk procedure called interspinous spacer (IS) or interspinous process device was invented to implant a spacer between the 2 adjacent spinal processes with lesions to prevent excessive dorsiflexion and to relieve symptoms induced by stenosis, such as Aperius, Coflex, DIAM, Wallis and X-stop, which could effectively unload the diseased lumbar motion segment and maintain mobility and structural elements.^[6,7]^ However, previous studies^[8,9]^ indicated that controversy remains about whether IS could produce better or worse outcomes than DS, and lacks strong evidence. Given the newly emerging evidence, we performed a meta-analysis to compare the effectiveness and safety of IS versus DS for lumbar spinal stenosis (LSS), and only randomized controlled trials (RCTs) were included in our meta-analysis to achieve high quality and credible results.

## 2. Materials and Methods

### 2.1. Search strategy

To make an exhaustive search of all relevant literature, 2 independent reviewers conducted a PRISMA (Preferred Reporting Items for Systematic Reviews and Meta-Analyses) compliant search of PubMed, EMBASE, and Cochrane Central Register of Controlled Trials (CENTRAL) for RCTs. The following search terms were used for the initial literature search: “(X-stop OR DIAM OR Wallis OR Coflex OR lumbar interspinous non-fusion technique OR IS) AND (DS OR decompression) AND Clinical Trial [ptyp].” We also reviewed the reference lists of retrieved studies and recent reviews. The retrieved results were last updated on July 30, 2023.

### 2.2. Criteria for selected trials

The PICOS (Patient/Problem, Intervention, Comparison, Outcome, Study design) strategy was utilized to guide the study selection process. We included studies that were eligible for the following criteria: (1) patients with symptomatic LSS; (2) the articles that provide IS versus DS as intervention treatments; (3) outcomes including at least one of the following data: surgical parameters (operation time, blood loss, hospital stay, dural violation, spinous process fracture, and reoperation), and clinical indexes; (4) RCTs were eligible. The exclusion criteria were as follows: (1) the researches were case reports, reviews, or observational studies. (2) The outcomes were descriptive or graphic with no numerical values; (3) the same data had been published repeatedly. Two reviewers independently selected the potentially qualified trials according to the inclusion and exclusion criteria. Any disagreement was resolved by discussion and conformity was reached.

### 2.3. Data extraction

Two independent reviewers extracted the data from eligible studies. If there were any disagreements, all of the authors discussed them until a consensus was achieved. The indispensable data extracted from eligible studies included published year, sample size, mean age, gender distribution, body mass index, number of levels, country, sample type, duration of follow-up, number of centers, and outcomes. The outcomes in this analysis included surgical parameters (operation time, blood loss, hospital stay, dural violation, spinous process fracture, and reoperation), and clinical indexes (visual analog scale leg pain [VAS lp], visual analog scale back pain [VAS bp], Oswestry Disability Index [ODI], Zurich Claudication Questionnaire Physical function [ZCQP], Zurich Claudication Questionnaire Symptom severity [ZCQS], foraminal height [FH], and disc height [DH]).

### 2.4. Quality assessment

The risks of bias in the RCTs were assessed independently by 2 of the authors by using the Cochrane Collaboration Risk of Bias Tool.^[10]^ The contents are 7 parts: random sequence generation (selection bias), allocation concealment (selection bias), blinding of participants and personnel (performance bias), blinding of outcome assessment data (detection bias), incomplete outcome data (attrition bias), selective reporting (reporting bias), and other bias. Each item was recorded as “high risk of bias,” “low risk of bias,” and “unclear risk of bias.” Any disagreement was discussed and resolved with a third independent author.

### 2.5. Statistical analysis

The weighted mean difference or standardized mean difference (SMD) and their 95% confidence interval (CI) were calculated for the continuous data, and the odds ratio (OR) and its 95% CI were calculated for the dichotomous data. The chi-square test and *I*-square test were used to assess the statistical heterogeneity.^[11]^ It demonstrated significant heterogeneity when a *P*-value of the chi-square test was <0.10 or *I*^2^ values exceeded 50%. A random-effects model was used when significant heterogeneity was observed among the included studies. Otherwise, a fixed-effects model was used for no significant heterogeneity. The possibility of publishing bias was not researched because of the limited number of included studies. This meta-analysis was performed by RevMan 5.3 software (Cochrane Collaboration, Copenhagen, Denmark). The statistically significant level was set at *P* < .05.

## 3. Results

### 3.1. Search results and quality assessment

**Figure 1.**
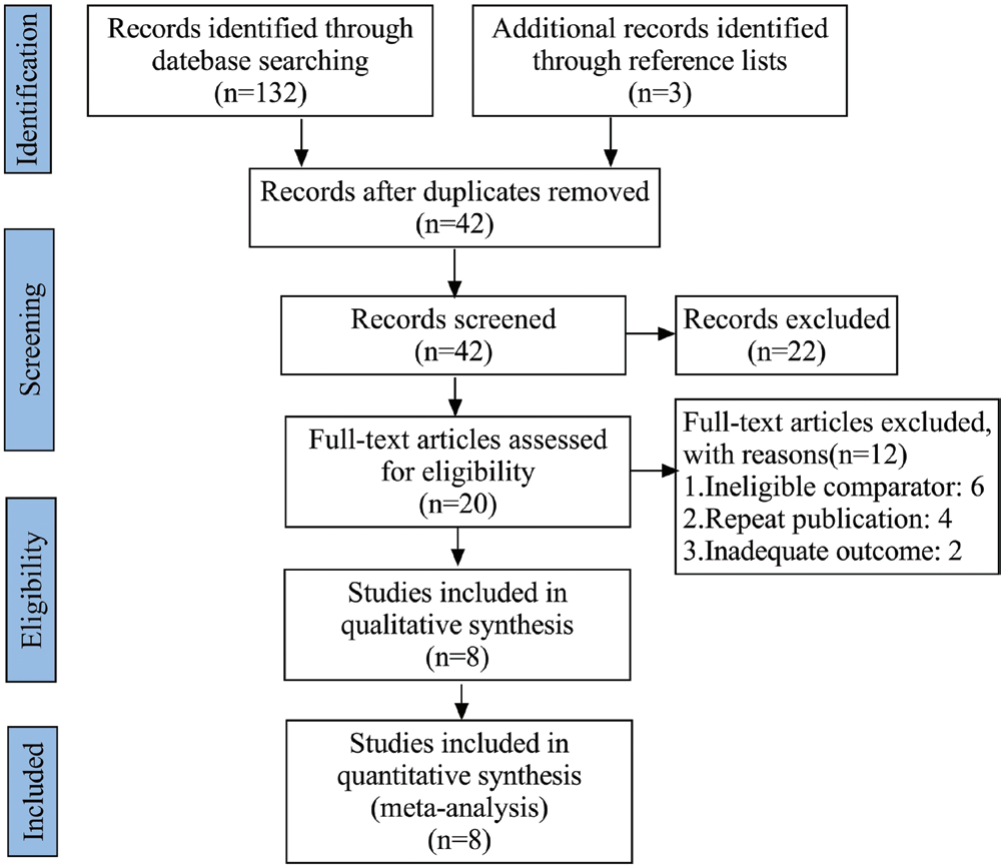
PRISMA Flow Diagram for Systematic Review and Meta-Analysis.

**Figure 2.**
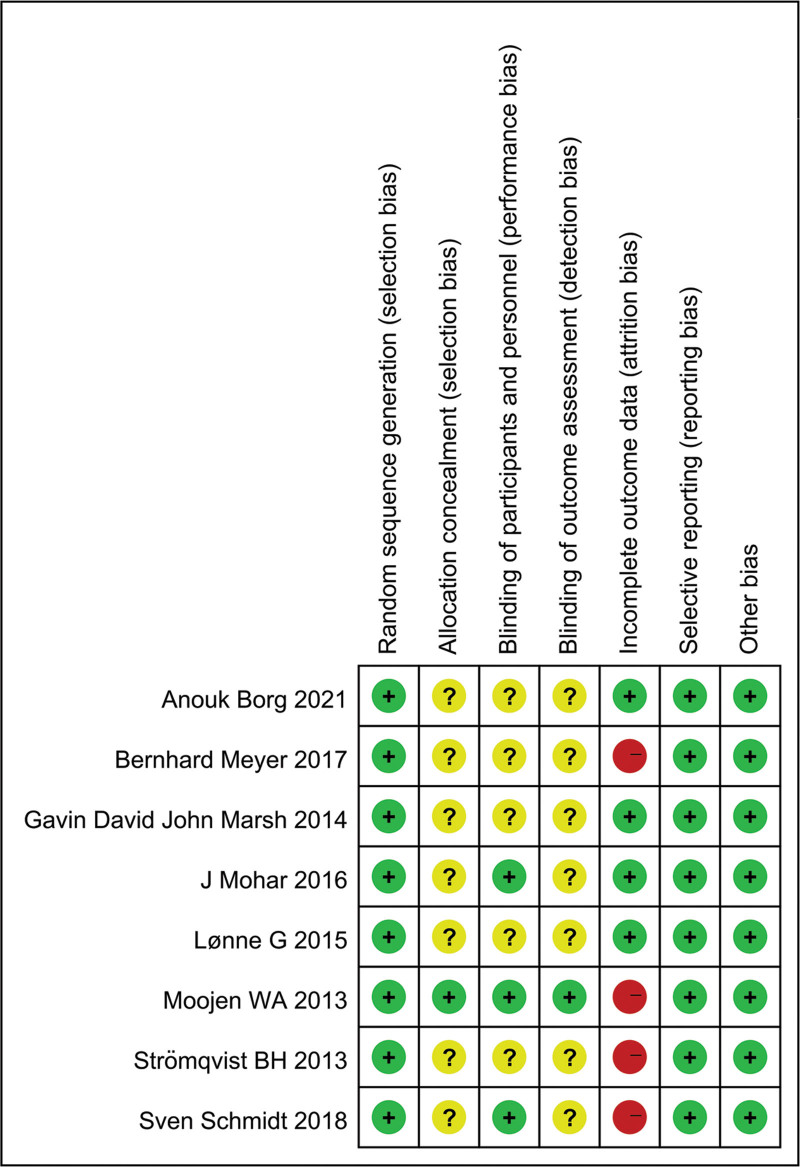
The risk of bias of the included studies was evaluated in our meta-analysis.

The electronic search originally identified 135 relevant studies. However, 115 were excluded after scrutiny of their titles or abstracts. The full publications were obtained for the leaving 20 studies. Furthermore, 12 papers were excluded for failing to meet the selection criteria, eventually leaving 8 eligible RCTs^[12–19]^ for analysis (Fig. 1). We recorded the characteristics of patients in the included studies (Table 1), as well as details of the studies and clinical outcomes of the studies (Table 2). The risk of bias was shown in Figure 2. All the 8 RCTs were high quality.

**Table 1:** Patient characteristics of the studies in the analysis.

Studies (year)	Sample size (IS/DS)	Mean age (years) (IS/DS)	Gender (proportion of male) (IS/DS)	BMI (kg/m^2^) (IS/DS)	Number of levels
Moojen (2013)	80/79	66/64	61%/47%	27/28	1, 2
Strömqvist (2013)	50/50	67/71	60%/52%	NR	1, 2
Marsh (2014)	30/30	59.6/56.4	36.7%/46.7	NR	1, 2
Lonne (2015)	40/41	67/67	42%/56%	28/28	1, 2
Mohar (2016)	6/6	49.2/51.2	66.7%/33.3%	27.4/25	1
Meyer (2017)	82/81	65/65	53%/49%	29/28	1, 2
Schmidt (2018)	115/115	68/68	42.7%/50%	29.1/29.3	1, 2
Borg (2021)	21/26	70/69	443%/35%	NR	1, 2, or 3

BMI = body mass index, DS = decompressive surgery, IS = interspinous spacer, NR = not reported.

**Table 2:** Characteristics and clinical outcomes of the studies in the analysis.

Studies (year)	Country	Device type	Number of centers	Follow-up (months)	Outcomes
Moojen (2013)	Netherlands	distraXion	5	12	VAS lp, VAS bp, reoperation, spinous process fracture, operation time, hospital stay
Strömqvist (2013)	Sweden	X-Stop	3	24	VAS lp, VAS bp, reoperation, dural violation, spinous process fracture
Marsh (2014)	Britain	Wallis	1	40	VAS bp, ODI
Lonne (2015)	Norway	X-Stop	6	24	ODI, ZCQP, ZCQS, reoperation, dural violation, hospital stay
Mohar (2016)	Slovenia	DIAM	1	6	FH, DH
Meyer (2017)	Multicenter	Aperius	19	24	VAS lp, VAS bp, ZCQP, ZCQS, reoperation, operation time, blood loss
Schmidt (2018)	Germany	Coflex	7	24	FH, DH, ODI, dural violation, operation time, blood loss
Borg (2021)	Britain	X-Stop	3	24	Operation time, dural violation, spinous process fracture

DH = disc height, FH = foraminal height, ODI = Oswestry Disability Index, VAS bp = visual analog scale back pain, VAS lp = visual analog scale leg pain, ZCQP = Zurich Claudication Questionnaire Physical function, ZCQS = Zurich Claudication Questionnaire Symptom severity.

## 4. Outcome analysis of surgical parameters

### 4.1. Operation time

**Figure 3.**
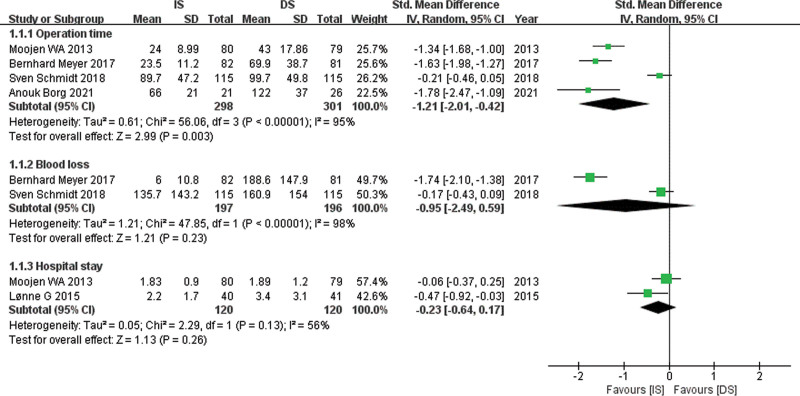
Forest plot comparing operation time, blood loss and hospital stay between IS and DS. DS = decompressive surgery, IS = interspinous spacer.

Relevant data for operation time was obtained from 4 studies^[12,17–19]^ with 298 patients in the IS group and 301 patients in the DS group. A random-effects model was used. The pooling of data in Figure 3 showed significantly less operation time in the IS group compared with the DS group (SMD, −1.21; 95% CI: −2.01 to −0.42, *P* = .003).

### 4.2. Blood loss

Relevant data for blood loss was obtained from 2 studies^[17,18]^ with 197 patients in the IS group and 196 patients in the DS group. A random-effects model was used. The pooled estimate of effect size for blood loss in Figure 3 showed no significant difference between the 2 groups (SMD, −0.95; 95% CI: −2.49 to 0.59, *P* = .23).

### 4.3. Hospital stay

Relevant data for hospital stay was obtained from 2 studies^[12,15]^ with 120 patients in the IS group and 120 patients in the DS group. A random-effects model was used. The pooled estimate of effect size for the hospital stay in Figure 3 showed no significant difference between the 2 groups (SMD, −0.23; 95% CI: −0,64 to 0.17, *P* = .26).

### 4.4. Dural violation

**Figure 4.**
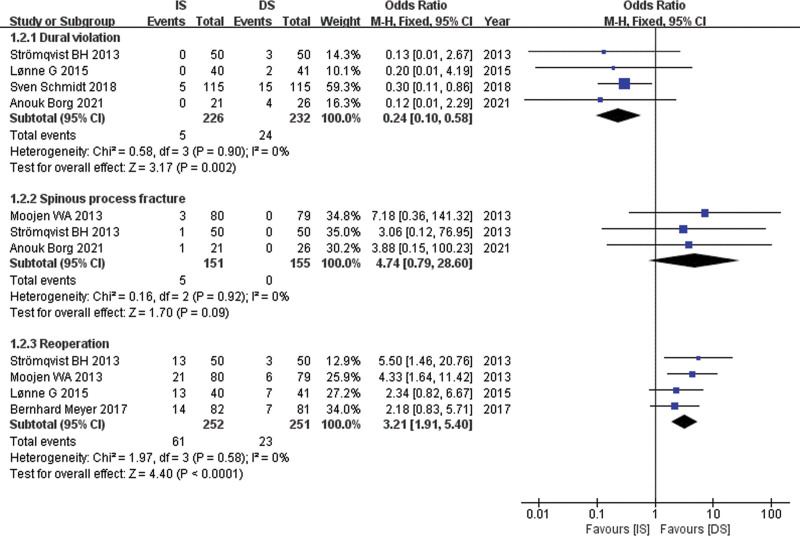
Forest plot comparing dural violation, spinous process fracture and reoperation between IS and DS. DS = decompressive surgery, IS = interspinous spacer.

Relevant data for dural violation was obtained from 4 studies^[13,15,18,19]^ with 226 patients in the IS group and 232 patients in the DS group. A fixed-effects model was used. The pooling of data in Figure 4 showed significantly less dural violation in the IS group compared with the DS group (OR, 0.24; 95% CI: 0.10 to 0.58, *P* = .002).

### 4.5. Spinous process fracture

Relevant data for spinous process fracture was obtained from 3 studies^[12,13,19]^ with 151 patients in the IS group and 155 patients in the DS group. A fixed-effects model was used. The pooled estimate of effect size for spinous process fracture in Figure 4 showed no significant difference between the 2 groups (OR, 4.74; 95% CI: 0.79 to 28.60, *P* = .09).

### 4.6. Reoperation

Relevant data for reoperation was obtained from 4 studies^[12,13,15,17]^ with 252 patients in the IS group and 251 patients in the DS group. A fixed-effects model was used. The pooling of data in Figure 4 showed a significantly more reoperation rate in the IS group compared with the DS group (OR, 3.21; 95% CI: 1.91 to 5.40, *P <* .0001).

## 5. Outcome analysis of clinical indexes

### 5.1. VAS pain

**Figure 5.**
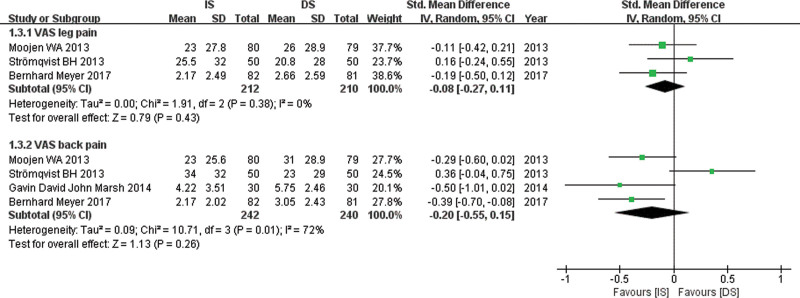
Forest plot comparing VAS score between IS and DS. DS = decompressive surgery, IS = interspinous spacer, VAS = visual analog scale.

Relevant data for VAS lp was obtained from 3 studies^[12,13,17]^ and VAS bp was obtained from 4 studies.^[12–14,17]^ A random-effects model was used. The pooled estimate of effect size for VAS lp (SMD, −0.08; 95% CI: −0.27 to 0.11, *P* = .43) and VAS bp (SMD, −0.20; 95% CI: −0.55 to 0.15, *P* = .26) in Figure 5 showed no significant difference between the 2 groups.

### 5.2. Oswestry Disability Index

**Figure 6.**
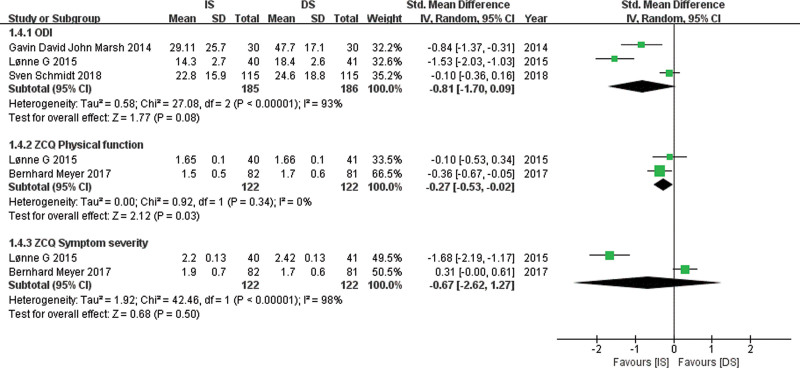
Forest plot comparing ODI, ZCQP, and ZCQS between IS and DS. DS = decompressive surgery, IS = interspinous spacer, ODI = Oswestry Disability Index, ZCQP = Zurich Claudication Questionnaire Physical function, ZCQS = Zurich Claudication Questionnaire Symptom severity.

Relevant data for ODI was obtained from 3 studies^[14,15,18]^ with 185 patients in the IS group and 186 patients in the DS group. A random-effects model was used. The pooled estimate of effect size for ODI in Figure 6 showed no significant difference between the 2 groups (SMD, −0.81; 95% CI: −1.70 to 0.09, *P* = .08).

### 5.3. Zurich Claudication Questionnaire Physical function and Symptom severity

Relevant data for ZCQP and ZCQS was obtained from 2 studies^[15,17]^ with 122 patients in the IS group and 122 patients in the DS group. A random-effects model was used. The pooled estimate of effect size for ZCQP in Figure 6 showed a significantly less ZCQP score in the IS group compared with the DS group (SMD, −0.27; 95% CI: −0.53 to −0.02, *P* = .03), and no significant difference for ZCQS between the 2 groups (SMD, −0.67; 95% CI: −2.62 to 1.27, *P* = .50).

### 5.4. Foraminal height and disc height

**Figure 7.**
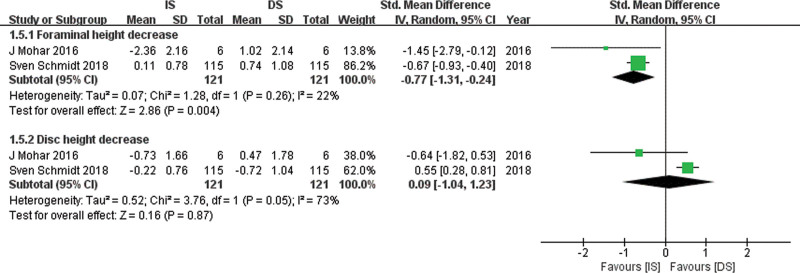
Forest plot comparing FH and DH between IS and DS. DH = disc height , DS = decompressive surgery, FH = foraminal height, IS = interspinous spacer.

Relevant data for FH and DH was obtained from 2 studies^[16,18]^ with 121 patients in the IS group and 121 patients in the DS group. A random-effects model was used. The pooled estimate of effect size for FH in Figure 7 showed significantly smaller FH decrease in the IS group compared with the DS group (SMD, −0.77; 95% CI: −1.31 to −0.24, *P* = .004), and no significant difference for DH decrease between the 2 groups (SMD, 0.09; 95% CI: −1.04 to 1.23, *P* = .87).

### 5.5. Sensitivity analysis

Sensitivity analysis investigated the influence of a single study on the overall outcome estimate by omitting 1 study in each turn. VAS bp score in the IS group was significantly less than that in the DS group when omitting the study by Stromqvist et al^[13]^ (*P* = .0004). ODI score in the IS group was significantly less than that in the DS group when omitting the study by Schmidt et al^[18]^ (*P* = .0006). The results of sensitivity analysis of other outcomes were not materially different compared with those of the original analysis. We analyzed that different versions of the rating scale and different IS types may yield different results in assessing disease symptoms or patient quality of life. The duration of follow-up may also impact study results, as disease symptoms or treatment effects can change over time.

## 6. Discussion

The IS is a new treatment concept for LSS, which enables the treatment of spinal pain and instability without fusing the involved spinal segment. However, a few systematic reviews and meta-analyses^[8,9,20,21]^ had reported that IS showed non-inferior efficacy and reliability for the treatment of LSS compared with DS. Non-RCT studies were included in the previous analyses, and other clinical outcomes and complications (such as DH decrease, FH decrease, spinous process fracture, and dural violation) of IS were not mentioned in these analyses. That is why we included only RCTs in our meta-analysis to determine whether IS was superior to DS for LSS.

We used operation time, blood loss, hospital stay, dural violation, spinous process fracture, and reoperation rate to describe the safety of IS and DS in treating LSS. The results of our meta-analysis demonstrated that IS had a significantly shorter operation time compared with the DS group. However, the pooled data showed that there were no significant differences between the 2 groups in blood loss and hospital stay. Dural violation is a familiar encounter during DS for LSS (6.5–9%).^[22–24]^ Desai et al^[25]^ indicated that dural violation during DS for LSS did not impact long-term outcomes in patients, although it considerably increased operation duration, blood loss, and hospital stay. In comparison to the DS group, the pooled data in our research revealed that IS can greatly lower the likelihood of dural violation. We summarized studies assessing the safety of IS and DS for LSS using multiple metrics. IS was found to have a significant advantage in terms of operative time, but did not differ significantly from DS in terms of bleeding and length of hospital stay. Dural rupture is a common complication of decompression surgery but has a limited impact on long-term outcomes, whereas IS reduces the risk of dural rupture. These findings have important implications for guiding treatment decisions.

The pooled data indicated that IS group had a higher rate of reoperation (such as removal of the implant, fusion, and extended DS) than the DS group, and no significant difference in the rate of spinous process fracture between both groups. Hao et al^[26]^ demonstrated that IS could lead to stress redistribution at the pars interarticularis, which could cause a potential spinous process fracture. David et al^[27]^ also indicated that these fractures were initially minimal or nondisplaced and the metallic wings of the devices often obscured fractures, and the unrecognized spinous process fracture may be responsible for a significant number of patients who experience reoperation after IS surgery.

VAS lp, VAS bp, ODI, ZCQP, and ZCQS, FH, and DH were used to describe the effects of IS and DS in treating LSS. In this analysis, we found that compared with DS, except for the less ZCQP score in IS group, the VAS score (both for leg and back), ODI, and ZCQS in the IS group were not significantly different. Therefore, we considered that the effect of IS was similar to that of the DS. Yusof et al^[28]^ identified a significant correlation between FH and DH. A decrease in FH may cause compression to the nerve root that crosses the foramen, and the increased DH resulted in greater FH and area, which can improve symptoms of nerve root pain.^[29]^ In our study, despite the fact that there were no significant variations in DH reduction between the 2 groups, patients treated with IS had a lower FH reduction than those treated with DS, which relied on the functional design of the IS. The results of the study showed that the effectiveness of IS and DS in the treatment of LSS was similar in most of the assessed metrics. However, for the effects on ZCQP score and FH, IS showed some advantages, which may be related to the functional design of IS. These results provide important information for clinicians and patients regarding treatment options for LSS, especially for those concerned with symptom relief and functional improvement.

There are several strengths in our study. First, only RCTs were included in our analysis to ensure high quality and credible results. Second, some clinical outcomes and complications that were not accessible in prior studies^[20,21]^ were included in our study. However, there are also several limitations in our study. First, the language of the included studies was limited to English, and studies in other languages were not considered for inclusion in this analysis. Second, the IS devices used in these studies were different types, which are potential sources of clinical heterogeneity. The use of IS in patients with severe LSS requires more caution, future studies should focus on indications for surgery in patients, complications, as well as the type and degree of spinal stenosis.

## 7. Conclusions

In summary, we considered that IS had similar effects with DS in hospital stay, blood loss, spinous process fracture, DH decrease, VAS score, ODI score, and ZCQS, and was better in some indices such as operation time, dural violation, ZCQP, and FH decrease than DS. However, due to the higher rate of reoperation in the IS group, we considered that both IS and DS were acceptable strategies for treating LSS. As a novel technique, further well-designed studies with longer-term follow-up are needed to evaluate the effectiveness and safety of IS.

## Author contributions

**Data curation:** Jia-Ju Che, Zhe Wang, Yu-Ming Chen, Bing Leng.

**Writing – original draft:** Jian-Hai Xin.

**Writing – review & editing:** Dalin Wang.
